# Integrated Transcriptomic and Metabolomic Analyses Reveal Adaptive Mechanisms of *Medicago sativa* Under Water Stress

**DOI:** 10.3390/plants15101531

**Published:** 2026-05-16

**Authors:** Yangyang Song, Nazi Niu, Yuanrong Wu, Qianqian Huo, Yuanyuan Qu, Linqiao Xi

**Affiliations:** 1 College of Animal Science and Technology, Tarim University, Alar 843300, China; ndymyys@163.com (Y.S.); 13635886155@163.com (Y.W.); 18742910910@163.com (Q.H.); 2 Key Laboratory of Livestock and Forage Resources Utilization Around Tarim, Ministry of Agriculture and Rural Affairs, Tarim University, Alar 843300, China; 3 College of Grassland Science and Technology, China Agricultural University, Beijing 100083, China; niunazi980219@163.com

**Keywords:** alfalfa, waterlogging stress, drought stress, transcriptome, metabolome

## Abstract

Water stress is a major abiotic constraint limiting the growth and productivity of alfalfa (*Medicago sativa* L.). To elucidate the adaptive mechanisms and identify key drought-tolerance genes, physiological measurements were integrated with multi-omics analyses of cultivar ‘Tamu 1’ under three water treatments: waterlogging (100% field water capacity), normal irrigation (80% FWC), and drought (light: 60% FWC, moderate: 40% FWC, severe: 20% FWC). Water stress markedly inhibited plant growth, induced oxidative stress, and reduced the photosynthetic capacity. Compared with waterlogging stress (DAMs: *n* = 71; DEGs: *n* = 313), drought stress resulted in a substantially greater number of differentially accumulated metabolites (DAMs, *n* = 1504) and differentially expressed genes (DEGs, *n* = 8006). Weighted gene co-expression network analysis (WGCNA) identified six key modules and ten hub genes associated with stress responses. Integrated transcriptomic and metabolomic analyses further revealed four major responsive pathways: starch and sucrose metabolism, phenylpropanoid and flavonoid metabolism, glutathione metabolism, and zeatin biosynthesis. Based on integrative criteria, including differential expression (|log_2_FC| ≥ 1, adjusted *p* < 0.05), WGCNA modules significantly associated with drought-related traits (R^2^ > 0.6), as well as functional annotation and protein–protein interaction (PPI) network topology, 28 candidate genes associated with drought tolerance were identified, of which six were further validated by quantitative real-time PCR (qRT-PCR). These findings highlight key metabolic pathways and regulatory modules underlying alfalfa responses to water stress and provide valuable candidate gene resources for improving drought tolerance.

## 1. Introduction

Water stress is a major factor that limits plant growth and productivity by disrupting physiological and metabolic processes due to soil water imbalance [1,2]. It can be classified into drought and waterlogging stress based on the soil water potential and root zone oxygen availability [3,4,5]. Water stress causes systemic damage to plants. Morphologically, it inhibits growth, resulting in plant dwarfism, leaf wilting and abscission [6,7]. Physiologically, it disrupts photosynthesis, with drought limiting CO_2_ uptake through stomatal closure, and waterlogging directly impairs photosystem II function [8,9]. Metabolically, it triggers a reactive oxygen species (ROS) burst and membrane lipid peroxidation and promotes the synthesis of osmotic adjustment substances (e.g., proline and soluble sugars) and antioxidant metabolites (e.g., flavonoids) [10,11]. To mitigate these injuries, plants have developed an active molecular regulatory network that enhances stress resistance by reprogramming gene expression and metabolic pathways [12,13].

In recent years, remarkable progress has been made in understanding the response mechanisms of alfalfa (*Medicago sativa* L.) to water stress. Several key regulatory factors have been identified. For instance, *MsCIPK2* enhances drought resistance by regulating root development and stomatal movement [14]; genes of the *CESA* superfamily exhibit differential expression patterns under drought and salt stress [15,16]; and the *ERF026* transcription factor acts as a molecular switch linking jasmonic acid signaling and growth regulation, thereby partially overcoming the traditional growth–defense trade-off paradigm [17,18]. However, most existing studies have focused on a single stress condition, such as drought or waterlogging [19,20]. In addition, these studies have largely relied on independent analyses of transcriptomic or metabolomic data. Consequently, the coordinated regulatory mechanisms underlying the integration of contrasting water stress signals within a single genotype remain poorly understood. Moreover, systematic analyses of carbon metabolism and energy allocation under combined or contrasting stress conditions are still limited [21,22]. Currently, plant abiotic stress research has increasingly shifted from single-omics approaches toward multi-omics integration and systems biology frameworks [23,24,25]. Integrated transcriptome and metabolome analyses, combined with systems biology tools such as weighted gene co-expression network analysis (WGCNA), enable the construction of gene–metabolite–phenotype association networks. These approaches facilitate the identification of core metabolic pathways and key hub genes with high precision [26,27,28]. Such strategies not only help overcome current research bottlenecks but also provide essential support for elucidating the adaptive mechanisms of alfalfa to water stress and accelerating the development of stress-tolerant elite germplasm resources [29,30].

The alfalfa cultivar ‘Tamu 1’ employed in this study is an elite germplasm with superior performance under arid and high-temperature conditions in southern Xinjiang and exhibits strong field adaptability. Nevertheless, the molecular regulatory mechanisms underlying the response to water stress remain to be systematically elucidated. Herein, we used ‘Tamu 1’ as the experimental material and established a continuous water gradient treatment ranging from waterlogging to severe drought. By integrating phenotypic, physiological, and biochemical assays, transcriptome sequencing, metabolomic profiling, and weighted gene co-expression network analysis (WGCNA), we aimed to systematically dissect the dynamic regulatory network governing alfalfa’s response to water stress, identify core metabolic pathways and key candidate genes, and thereby provide a theoretical foundation and genetic resources for drought−resistant alfalfa breeding and efficient water management practices.

## 2. Results

### 2.1. Effects of Water Stress on Phenotypic and Physiological Traits of Alfalfa

**Figure 1:**
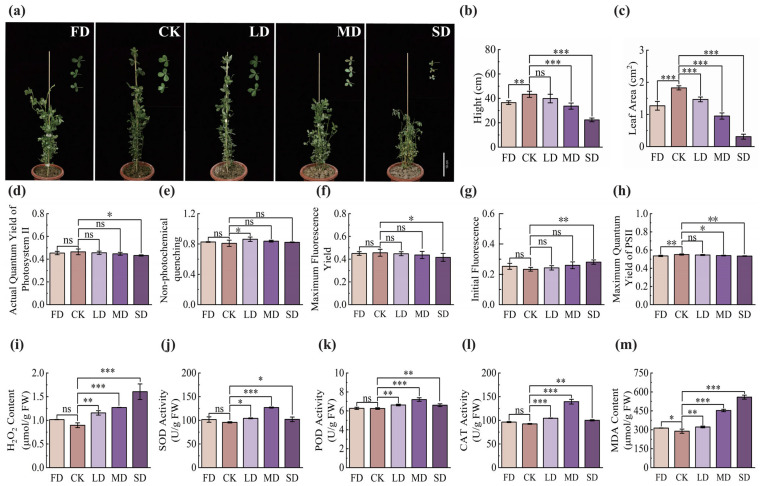
Phenotypic and physiological responses of alfalfa under different water stress treatments. (**a**) Phenotypes. (**b**) Plant height, scale bar—refers to subfigure a. (**c**) Leaf area. (**d**) Actual quantum yield of photosystem II (ΦPSII). (**e**) Non-photochemical quenching (qN). (**f**) Maximum fluorescence yield (Fm’). (**g**) Initial fluorescence (Fo). (**h**) Maximum quantum yield of PSII (Fv/Fm). (**i**) H_2_O_2_ content. (**j**) Superoxide dismutase (SOD) activity. (**k**) Peroxidase (POD) activity. (**l**) Catalase (CAT) activity. (**m**) MDA content. Plants were subjected to a water gradient: waterlogging (FD, 100 ± 5% FWC), control (CK, 80 ± 5% FWC), light drought (LD, 60 ± 5% FWC), moderate drought (MD, 40 ± 5% FWC), and severe drought (SD, 20 ± 5% FWC). Data are presented as mean ± SD (*n* = 3), with error bars representing the standard deviation. Different asterisks above the bars indicate significant differences among treatments (one−way ANOVA followed by Fisher’s LSD test, *p* < 0.05). The symbols *, **, and *** denote significance levels at *p* < 0.05, *p* < 0.01, and *p* < 0.001, respectively; “ns” indicates no significant difference.

Water stress markedly altered phenotypic and physiological traits of ‘Tamu 1’ alfalfa (Figure 1). Drought induced progressive wilting (Figure 1a) and significantly reduced plant height and leaf area versus CK (*p* < 0.05). Waterlogging (FD) similarly decreased these traits (*p* < 0.05) (Figure 1b,c). Photosynthetic performance was markedly influenced by the water stress (Figure 1d–h). Under drought conditions, increasing stress severity led to progressive declines in ΦPSII, qN, Fm′, and Fv/Fm, accompanied by an increase in Fo. The reduction in ΦPSII suggests decreased photosynthetic electron transport efficiency, indicating impaired PSII function. The decline in Fv/Fm further confirms the occurrence of photoinhibition, while the increase in Fo reflects damage to PSII reaction centers or disruption of energy transfer. The decrease in qN indicates a reduced capacity for non-photochemical energy dissipation or altered energy allocation under stress conditions. Significant differences between the severe drought (SD) treatment and CK were observed for ΦPSII, Fm′, and Fo (*p* < 0.05). In contrast, under FD, ΦPSII, qN, Fm′, and Fo increased relative to CK, whereas Fv/Fm was significantly reduced (*p* < 0.05). The increase in ΦPSII under waterlogging may reflect a short-term compensatory enhancement of electron transport efficiency or altered energy allocation, despite overall PSII damage as indicated by reduced Fv/Fm (Figure 1d–h). Water stress also induced substantial changes in antioxidant enzyme activities and oxidative stress markers (Figure 1i–m). Under drought stress, the H_2_O_2_ and MDA levels increased with increasing stress intensity. The activities of POD, SOD, and CAT initially increased and then declined under severe drought conditions, although they remained significantly higher than those in the CK (*p* < 0.05). In the waterlogging treatment, SOD, POD, and CAT activities, as well as H_2_O_2_ and MDA levels, were all higher than those of CK, with MDA showing a significant difference (*p* < 0.05).

### 2.2. Differential Metabolomic Analysis Under Water Stress

**Figure 2:**
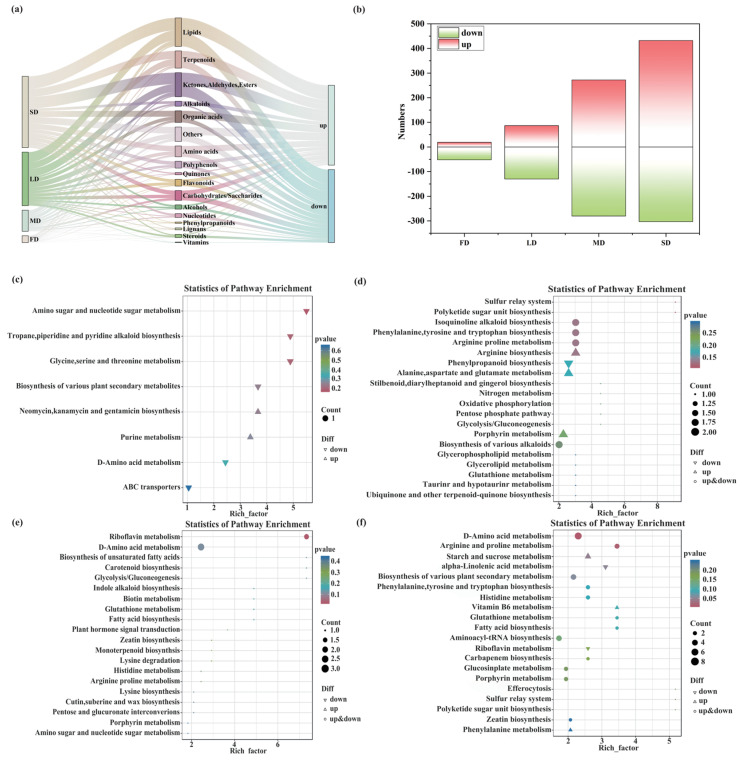
Metabolomic analysis of alfalfa leaves under water stress conditions. (**a**) Sankey diagram showing the classification and distribution of differential metabolites in alfalfa leaves. Different colors represent different classes of metabolites, and the width of each connection represents the relative abundance of metabolites in each class. (**b**) Number of upregulated and downregulated differentially accumulated metabolites (DAMs) under each water stress treatment. (**c**–**f**) KEGG pathway enrichment analysis of DAMs under different stress treatments: (**c**) FD, (**d**) LD, (**e**) MD, and (**f**) SD. In the bubble plots, the *x*-axis represents the enrichment factor, and the *y*-axis represents the pathway name. The bubble size indicates the number of DAMs enriched in each pathway, and the color represents the adjusted *p*−value (red indicates significant differences, while blue indicates non-significant differences; darker colors represent higher statistical significance).

Metabolomic profiling was conducted using ultra-performance liquid chromatography coupled with tandem mass spectrometry (UPLC-ESI-MS/MS). Principal component analysis (PCA) and hierarchical clustering analysis (HCA) demonstrated a clear separation between the treatment groups and the CK, with tight clustering of biological replicates, indicating high data reliability and reproducibility (Supplementary Figure S1). A total of 1575 metabolites belonging to 18 chemical classes were identified in the alfalfa leaves (Figure 2a). The number of differentially accumulated metabolites (DAMs) showed a strong dependence on both stress type and intensity. Only 71 DAMs were detected under waterlogging stress (FD), whereas drought stress induced substantially more DAMs: 217 under light drought (LD), 552 under moderate drought (MD), and 735 under severe drought (SD) (Figure 2b). Drought thus caused substantially greater metabolic perturbation than waterlogging (*χ^2^* = 703.25, *p* < 0.001). Moreover, DAM numbers increased progressively with drought severity, indicating a dose−dependent metabolic response.

Distinct metabolite classes were enriched under different water-stress conditions. Under water stress, differential metabolites were predominantly enriched in lipids, ketones, aldehydes, esters, terpenoids, and organic acids (enrichment factor = 0.53, *p* < 0.05). The total number of DAMs in the drought treatments was 797, which was approximately 18.1 times that of the FD group. KEGG enrichment analysis further revealed stress-specific metabolic responses. DAMs in FD were mainly enriched in pathways related to alkaloid biosynthesis (e.g., ko00960, ko00996), carbohydrate metabolism (e.g., ko00010, ko00020, ko00052), amino acid metabolism (e.g., ko00250, ko00260, ko00330), and ABC transport (ko02010). In the LD treatment, the significantly enriched pathways included flavonoid biosynthesis (ko00941/ko00944), isoflavonoid biosynthesis (ko00943), and lipid metabolism. Under MD conditions, DAMs were mainly enriched in galactose (ko00052) and amino acid metabolism pathways. In addition to several shared pathways observed under other drought treatments, SD showed strong enrichment in amino acid metabolism pathways (*p* < 0.05) (Figure 2c–f).

Notably, all water stress treatments enriched pathways involved in secondary metabolite biosynthesis and amino acid metabolism. Five metabolites, including aperuloside, consistently and significantly accumulated across all treatments (*p* < 0.05) (Supplementary Figure S2). As a key iridoid glycoside secondary metabolite, asperuloside likely contributes to antioxidant defense and cellular protection by modulating reactive oxygen species (ROS) scavenging and activating the Nrf2/HO-1 signaling pathway, thereby helping maintain redox homeostasis under water stress conditions. Terpenoids function as core broad−spectrum defensive substances to eliminate excess ROS and mitigate oxidative injury; flavonoids serve as critical non-enzymatic antioxidants to strengthen antioxidant capacity; soluble sugars and carbohydrate derivatives participate in osmotic adjustment, stabilizing cell structure and improving stress adaptability. These findings suggest that alfalfa adopts distinct metabolic strategies to cope with different types of water stress. Waterlogging preferentially activates the synthesis of broad-spectrum defensive metabolites, such as terpenoids, whereas drought, particularly severe drought, enhances metabolic processes associated with osmotic adjustment and antioxidant defense.

### 2.3. Differential Transcriptome Analysis Under Water Stress

Transcriptome sequencing of the alfalfa cultivar ‘Tamu 1’ generated 104.40 Gb of clean data, with Q30 values exceeding 93.05%, indicating high sequencing quality (Supplementary Table S1). Principal component analysis revealed a clear separation between samples from different water stress treatments and the control at the transcriptional level (Supplementary Figure S3a). Correlation analysis and hierarchical clustering further confirmed high reproducibility among biological replicates and distinct gene expression patterns among treatments (Supplementary Figure S3b,c).

**Figure 3:**
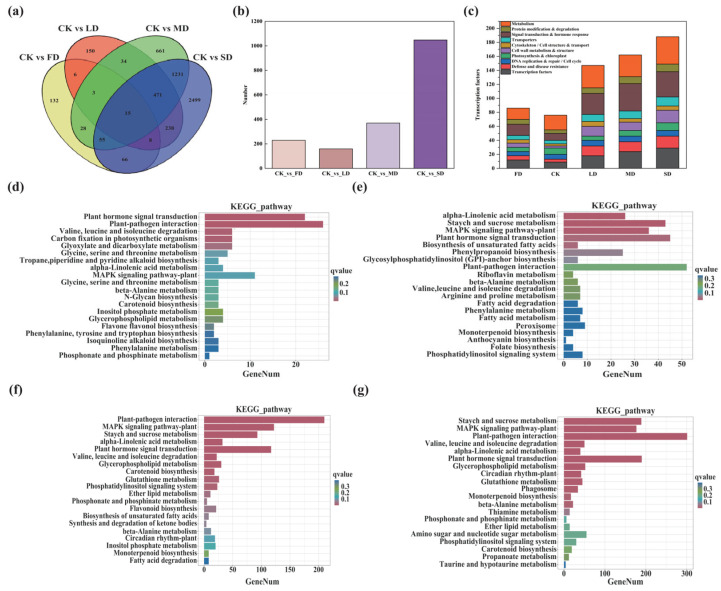
Transcriptomic responses of alfalfa leaves to different water stress treatments. (**a**) Venn diagram illustrating the overlap of differentially expressed genes (DEGs) among the four water stress treatments compared with CK. (**b**) Number of upregulated DEGs identified in each treatment. (**c**) Functional classification of significantly enriched KEGG categories among upregulated DEGs. The height of each colored bar represents the number of genes enriched in the corresponding category. (**d**–**g**) KEGG pathway enrichment analysis of upregulated DEGs under different water stress treatments: (**d**) FD, (**e**) LD, (**f**) MD, and (**g**) SD. The length of each bar represents the number of enriched DEGs, and the color indicates the adjusted *p*−value (red indicates significant enrichment, while blue indicates non-significant enrichment).

Compared with CK, the number of differentially expressed genes (DEGs) increased markedly under all water stress conditions. Notably, drought treatments induced substantially more DEGs than waterlogging treatments (FD = 313, LD = 925, MD = 2498, SD = 4583; Figure 3a), suggesting that drought elicits a stronger transcriptional response in alfalfa. Among these DEGs, 229, 159, 370, and 1048 genes were upregulated in the FD, LD, MD, and SD groups, respectively (Figure 3b). This difference was statistically significant (*χ^2^* = 1101.92, *p* < 0.001), indicating a strong dose−dependent increase in transcriptional reprogramming with increasing drought severity. KEGG functional enrichment analysis showed that the upregulated DEGs were primarily associated with transcriptional regulation (e.g., plant hormone signal transduction, ko04075), defense responses (e.g., plant−pathogen interaction, ko04626), and signal transduction pathways (e.g., MAPK signaling pathway−plant, ko04016; Figure 3c). Genes involved in signal transduction and plant hormone signaling were particularly responsive to water stress. Specifically, 16, 30, 39, and 36 upregulated DEGs belonging to these functional categories were identified in the FD, LD, MD, and SD groups, respectively. Further pathway enrichment analysis revealed distinct regulatory patterns under different stress conditions (Figure 3d). In the FD treatment, the most significantly enriched pathways were mainly associated with signal transduction. Under LD conditions, the enrichment shifted toward pathways related to cell wall metabolism. In the MD treatment group, additional enrichment was observed in pathways involving transcription factor regulation, defense responses, and diverse metabolic processes. In the SD treatment, all core pathways identified under MD were retained, whereas the enrichment levels of metabolic and defense−related pathways were further enhanced. These results suggest that alfalfa exhibits stage−specific and targeted transcriptional responses to water stress. Several pathways were consistently enriched across all treatments, particularly starch and sucrose metabolism (ko00500), flavonoid biosynthesis (ko00941), and plant hormone signal transduction (ko04075), indicating their central roles in the alfalfa water stress response.

### 2.4. WGCNA and Hub Genes Associated with Core Metabolites

**Figure 4:**
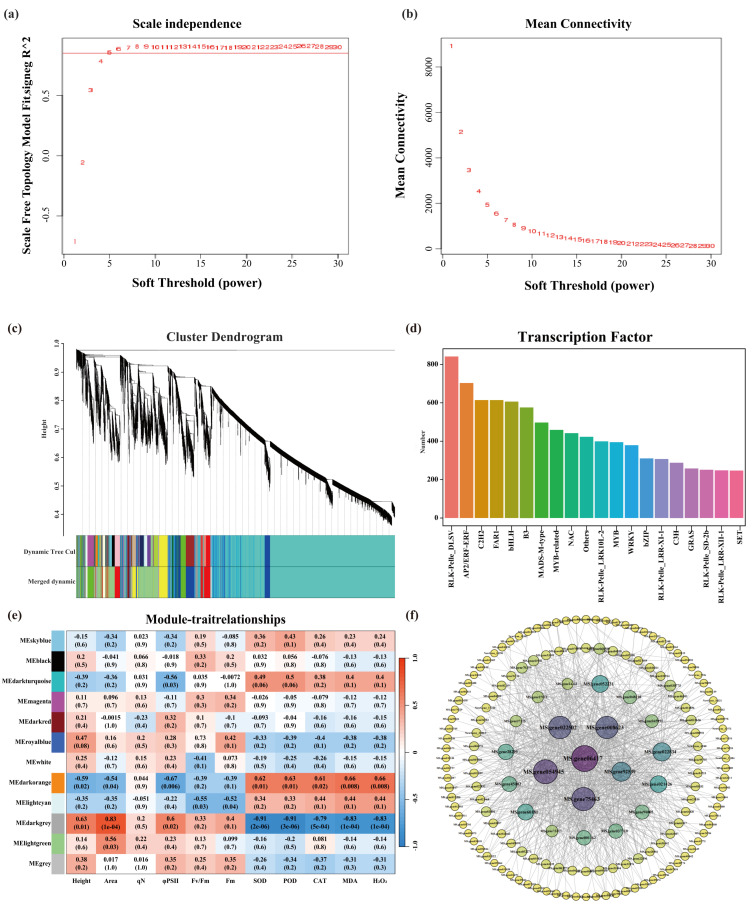
Weighted gene co−expression network analysis (WGCNA) and identification of hub genes. (**a**) Scale independence plot, showing the correlation between scale−free topology fit index and soft−thresholding power. (**b**) Mean connectivity plot, displaying the relationship between mean connectivity and soft−thresholding power. (**c**) Cluster dendrogram of gene co−expression modules identified by WGCNA, with different colors representing distinct gene modules. (**d**) Distribution of predicted transcription factor (TF) families across modules. (**e**) Heatmap showing the correlations between module eigengenes and physiological traits. Each row represents a gene module, and each column represents a physiological trait. The color scale indicates the correlation coefficient. (**f**) Co−expression network of hub genes within the key module associated with drought stress response.

Weighted gene co−expression network analysis (WGCNA) was performed to identify the gene modules associated with key metabolites under water stress. The relative abundance of differentially accumulated metabolites (DAMs) under each stress condition was used as trait data for network construction. A soft−thresholding power of β = 5 was selected to satisfy the scale−free topology criterion (R^2^ > 0.85), ensuring reliable co−expression relationships (Figure 4a,b). Hierarchical clustering combined with dynamic tree cutting identified 15 co−expression modules, each consisting of genes with highly correlated expression patterns (Figure 4c). Further analysis revealed stress−specific distribution patterns among the modules. Genes responsive to FD were mainly enriched in the yellow module, whereas genes associated with LD were primarily distributed in the purple, light−yellow, and blue modules. Genes responsive to MD were mainly enriched in the dark−green module, whereas those associated with SD were predominantly located in the grey60 module (Supplementary Figure S4a). These results indicate that increasing stress intensity activates distinct transcriptional modules. Transcription factor (TF) prediction identified 19 TF families in all modules. Among them, RLK−Pelle_DLSV, AP2/ERF−ERF, C2H2, FAR1, and bHLH were the most abundant, suggesting their important roles in water stress regulation (Figure 4d). Notably, the TF family distribution varied across stress conditions: FD treatment was enriched with AP2/ERF, bHLH, and HD−Zip TFs; LD treatment was dominated by AP2/ERF, WRKY, and NAC families; MD treatment showed enrichment of bHLH, GRAS, and HD−Zip TFs; whereas SD treatment mainly contained GRAS, HD−Zip, and RR−type TFs (Supplementary Table S2). These results suggest that different TF families may perform specialized regulatory functions under varying water stress levels.

Pearson correlation analysis between the module eigengenes (MEs) and physiological traits revealed significant associations between specific modules and stress responses. The dark−orange module showed strong positive correlations with antioxidant indicators, including H_2_O_2_, SOD, POD, CAT, and MDA (R^2^ = 0.66, 0.62, 0.63, 0.61, and 0.66, respectively; *p* < 0.05), whereas the dark−gray module exhibited positive correlations with growth−related traits, such as plant height, leaf area, and chlorophyll fluorescence parameters (Figure 4e). These results suggest that the dark−orange module may be involved in regulating antioxidant defense, whereas the dark−green module contributes to maintaining plant growth and photosynthetic capacity under water stress.

To identify key regulatory genes, hub genes within the dark−orange module were ranked according to node connectivity (degree value), and the gene interaction network was visualized using Cytoscape (Figure 4f). The top ten hub genes could be functionally categorized into three main groups. Phenylpropanoid/lignin: caffeic acid 3−O−methyltransferase (*COMT*, *MS.gene06417*), cinnamoyl−CoA reductase 1 (*CCR1*, *MS.gene008623*), and (R,S)−reticuline 7−O−methyltransferase (*ROMT*, *MS.gene045991*); osmotic adjustment: Δ1−pyrroline−5−carboxylate synthase isoform X2 (*P5CS2*, *MS.gene92539*), D−pinitol dehydrogenase (*PDH*, *MS.gene45002*), and inositol−3−phosphate synthase (*IPS*, *MS.gene70359*); stress signaling and degradation: PLAT domain−containing protein 3 (*PLAT3*, *MS.gene75463*), UV−B−induced protein (*UVB*−*IP*, *MS.gene022834*), CBL−interacting serine/threonine−protein kinase 6 (*CIPK6*, *MS.gene60181*), and cysteine protease 15A (*CP15A*, *MS.gene27275*). These genes likely play central roles in the regulatory network underlying alfalfa adaptation to water stress conditions.

### 2.5. Integrated Transcriptome and Metabolome Analysis

KEGG pathway enrichment analysis revealed that metabolic reprogramming induced by different water stress conditions was inherently distinct. Specifically, FD uniquely enriched pathways related to alkaloid biosynthesis, transmembrane transport, and amino acid and nucleotide metabolism. In contrast, the three drought treatments constitutively activated a core set of metabolic response networks, with significant enrichment observed in four key pathways: carbohydrate metabolism, glutathione metabolism, flavonoid metabolism and zeatin biosynthesis. These pathways collectively constitute the fundamental metabolic framework underlying alfalfa adaptation to drought stress. By integrating WGCNA, we identified the dark−orange module as a key module associated with the stress response. Focusing on its hub genes, we elucidated the stress tolerance mechanisms mediated by the expression patterns of genes and the dynamic changes in metabolites within these four pathways.

**Figure 5:**
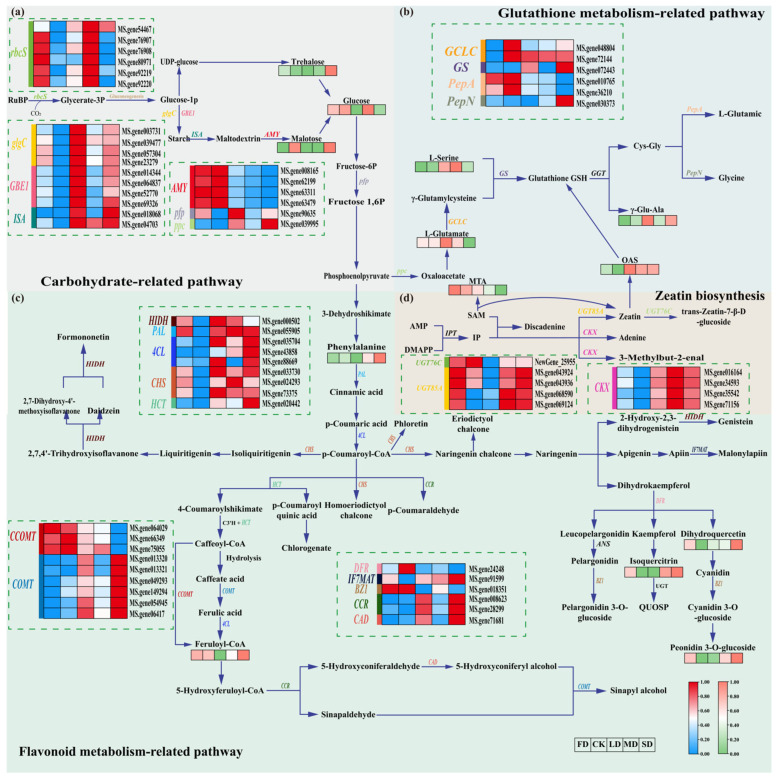
Comparative analysis of gene expression and metabolite levels in alfalfa leaves across metabolic pathways associated with carbohydrates, glutathione, flavonoids and zeatin. (**a**) Carbohydrate metabolic pathway. (**b**) Glutathione metabolism pathway. (**c**) Flavonoid metabolism pathway. (**d**) zeatin metabolism pathway. In the heatmap, each row corresponds to a gene or metabolite, and the five columns represent different water stress treatments. Red and blue indicate gene expression levels, whereas orange and green denote relative metabolite abundance. Enzyme abbreviations: *rbcS*, ribulose bisphosphate carboxylase small chain; *glgC*, glucose−1−phosphate adenylyltransferase; *GBE1*, 1,4−alpha−glucan−branching enzyme; *ISA*, isoamylase; *AMY*, α−amylase; *pfp*, diphosphate−dependent phosphofructokinase; *ppc*, phosphoenolpyruvate carboxylase; *GCLC*, glutamate−cysteine ligase; *GS*, glutathione synthase; *PepA*, leucyl aminopeptidase; *PepN*, aminopeptidase N; *CHS*, chalcone synthase; *HCT*, shikimate O−hydroxycinnamoyltransferase; *DFR*, dihydroflavonol 4−reductase; *CCOMT*, caffeoyl−CoA O−methyltransferase; *HIDH*, 2−hydroxyisoflavanone dehydratase; *IF7MAT*, isoflavone 7−O−glucoside-6′′−O−malonyltransferase; *BZ1*, anthocyanidin 3−O−glucosyltransferase; *PAL*, phenylalanine ammonia−lyase; *4CL*, 4−coumarate-CoA ligase; *CCR*, cinnamoyl−CoA reductase; *CAD*, cinnamyl−alcohol dehydrogenase; *COMT*, caffeic acid O−methyltransferase; *CKX*, cytokinin dehydrogenase; *UGT85A*, UDP−glucosyltransferase 85A.

The analysis of differentially expressed genes and metabolites involved in the starch and sucrose metabolism (ko00500), fructose and mannose metabolism (ko00051), and carbon metabolism (ko01200) pathways revealed that water stress universally induced carbohydrate accumulation in alfalfa (Figure 5a). *ISA* was identified as a core regulatory gene commonly responsive to both drought and waterlogging stresses. It encodes an isoamylase that mediates starch synthesis, degradation, and remodeling, thereby modulating carbon metabolic flux to supply essential carbon skeletons and energy for stress adaptation. This *ISA*−mediated carbon remodeling, together with the proline biosynthesis pathway regulated by *P5CS2*, synergistically enhances osmotic adjustment capacity in alfalfa through the co−accumulation of carbohydrates and proline (Supplementary Tables S3–S5).

The glutathione metabolism pathway (ko00480) constitutes a core defense system in alfalfa for scavenging ROS and maintaining cellular redox balance (Figure 5b). Glutathione Synthetase (*GS*), a core regulatory gene commonly upregulated under water stress conditions, drives the biosynthesis of glutathione (GSH). This transcriptional activation is accompanied by a significant accumulation of precursor metabolites, such as L−Glutamate and L−γ−Glutamylcysteine, in response to drought treatment. Furthermore, glutathione metabolism was closely associated with stress signal transduction mediated by *CIPK6*, a hub gene in the dark−orange module. Together, they participate in the antioxidant protection mechanism, a finding corroborated by WGCNA, which revealed a significant positive correlation between the dark−orange module and antioxidant enzyme activities.

As a core functional branch of phenylpropanoid metabolism (ko00940), flavonoid−related biosynthetic pathways represent a pivotal hub for the regulation of secondary metabolism (Figure 5c). Integrated analysis of the expression profiles of DAMs and DEGs involved in the phenylpropanoid (ko00940), flavonoid (MAP00941), anthocyanin (ko00942), isoflavonoid (MAP00943), and flavone and flavonol (MAP00944) biosynthesis pathways revealed that phenylalanine ammonia−lyase (*PAL*) was generally upregulated under water stress, redirecting metabolic flux towards secondary metabolic branches. Two key hub genes in the dark−orange module, *COMT* and *CCR1*, were significantly upregulated under drought stress, directly promoting the synthesis of monolignols and reinforcing the cell wall architecture. Concurrently, chalcone synthase (*CHS*) was markedly induced, leading to the significant accumulation of its downstream products, quercetin and anthocyanins, under MD and SD conditions, thereby enhancing ROS scavenging capacity. Furthermore, these processes act in concert with the functions of another dark−orange module hub gene, *PLAT3*, which is potentially involved in membrane lipid modification and stress response. Together, they orchestrate a comprehensive secondary metabolic network underpinning both cell wall fortification and antioxidant defenses.

Zeatin biosynthesis (ko00908) is a core pathway involved in the homeostatic regulation of cytokinins (Figure 5d). Under drought stress, the significant upregulation of cytokinin oxidase/dehydrogenase (*CKX*) accelerates the degradation of zeatin, indicating that plants adapt to adverse conditions by reducing the levels of growth−promoting hormones. Concurrently, the significant accumulation of the differential metabolite O−acetylserine (OAS) provides a substrate for L−cysteine synthesis and the downstream GSH−mediated antioxidant pathway, thereby facilitating crosstalk between hormone signaling and antioxidant metabolism.

### 2.6. Validation of RNA−Seq Data by qRT−PCR

**Figure 6:**
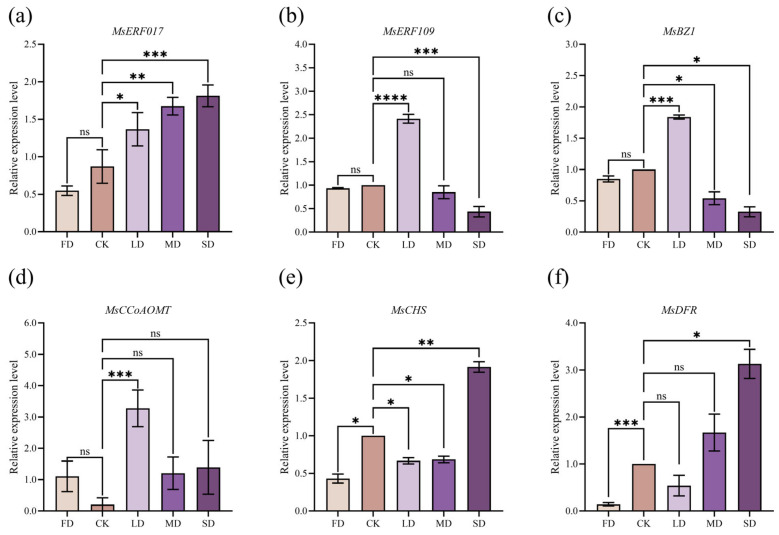
Validation of transcriptome data using qRT−PCR. The relative expression levels of six genes under different water stress treatments were analyzed using qRT−PCR. (**a**) *MsERF017*; (**b**) *MsERF109*; (**c**) *MsBZ1*; (**d**) *MsCCoAOMT*; (**e**) *MsCHS*; (**f**) *MsDFR*. The *x*-axis represents the different treatments, and the *y*−axis indicates the relative expression levels of each gene. Error bars represent the standard deviation. Asterisks indicate significant differences (* *p* < 0.05, ** *p* < 0.01, *** *p* < 0.001, and **** *p* < 0.0001; ns, not significant). Abbreviations: *MsERF017*: ethylene−responsive transcription factor ERF017; *MsERF109*: ethylene−responsive transcription factor ERF109; *MsBZ1*: anthocyanidin 3−O−glucosyltransferase; *MsCCoAOMT*: caffeoyl−CoA O−methyltransferase; *MsCHS*: chalcone synthase; *MsDFR*: dihydroflavonol 4−reductase.

Based on the aforementioned analyses, the DAMs under water stress conditions were significantly enriched in flavonoid-related biosynthetic pathways, which are known to be regulated by the corresponding transcription factors. To validate the reliability of the transcriptomic data, we performed quantitative real−time PCR (qRT−PCR) analysis of key structural genes (*MsCHS*, *MsDFR*, *MsBZ1*, and *MsCCoAOMT*) involved in flavonoid biosynthesis, as well as ERF transcription factor family members (*MsERF017* and *MsERF109*) that exhibited high abundance in the WGCNA and are potentially implicated in the transcriptional regulation of flavonoid metabolism (Figure 6). The qRT−PCR results demonstrated expression trends consistent with the RNA−seq data: *MsCHS*, *MsDFR*, *MsCCoAOMT*, and *MsERF017* were upregulated, whereas *MsBZ1* and *MsERF109* were downregulated, all correlating well with the transcriptomic profiles. This mixed up and dowregulation of pathway genes reflects a dynamic metabolic balance: some genes promote substrate synthesis, while others catalyze turnover or downstream modification, jointly maintaining homeostasis under water stress. Therefore, the transcriptomic data generated in this study are reliable and provide a robust foundation for subsequent investigations into the regulation of flavonoid biosynthetic pathways and functional characterization of ERF transcription factors.

## 3. Discussion

Water stress profoundly impairs the growth and development of ‘Tamu 1’ alfalfa. Our study corroborates previous findings that drought induces wilting and growth suppression, while waterlogging primarily restricts plant height and leaf area expansion [31,32]. These phenotypic alterations are intrinsically linked to disruptions in water balance and cellular turgor. More critically, our physiological data reveal that the underlying mechanisms of photosynthetic damage differ between stress types. Drought progressively diminished ΦPSII, qN, and Fv/Fm, indicating a cumulative impairment of PSII photochemical efficiency and photoprotective capacity [7,33]. Conversely, waterlogging triggered a specific decline in Fv/Fm alongside transient increases in ΦPSII and qN. This pattern suggests an initial, hypoxia−driven disruption of PSII reaction centers, potentially compensated by altered energy partitioning−a response distinct from the chronic photoinhibition observed under drought [34,35].

The antioxidant system also exhibited stress−specific modulation. Under drought, activities of POD, SOD, and CAT displayed a biphasic response, peaking under moderate stress before declining under severe conditions, coinciding with escalated H_2_O_2_ and MDA levels. This aligns with the concept that antioxidant defenses can be overwhelmed under intense, prolonged drought [36,37]. In contrast, waterlogging induced a sustained elevation in all measured antioxidant enzymes and oxidative markers, reflecting a continuous battle against hypoxia−induced ROS generation [38]. These differential physiological signatures underscore that alfalfa deploys distinct tactical responses: a phased defense that may falter under extreme drought versus a constitutive, broad−spectrum activation under waterlogging.

Moving beyond descriptive cataloging, our integrated transcriptomic and metabolomic analysis establishes causative links within the stress response cascade, addressing the core mechanistic gap. We identified and validated four cornerstone pathways whose coordinated rewiring forms the mechanistic backbone of adaptation.

Carbohydrate metabolism serves as a primary source of osmolytes and energy under water deficit [39,40]. Our data demonstrate that this is not a passive accumulation but an actively regulated process. The upstream genetic trigger was the consistent induction of *ISA* (isoamylase) across all stress conditions. *ISA* facilitates starch debranching and remobilization [41,42]. This specific gene action directly led to the intermediate metabolic outcome: the significant accumulation of soluble sugars (trehalose, maltose). Crucially, this metabolic shift propelled the downstream physiological adaptation: enhanced osmotic potential, which contributes to turgor maintenance and ROS mitigation under drought [43,44,45]. Furthermore, the synergy between *ISA*−mediated sugar release and the *P5CS2*−driven proline biosynthesis (a hub gene from the stress−correlated dark−orange module) presents a compelling example of multi−gene orchestration achieving a unified physiological goal−osmotic homeostasis.

Glutathione (GSH) metabolism is central to redox homeostasis [46]. Our analysis reveals its precise, intensity−dependent regulation. Under mild stress (LD, FD), the core regulatory genes *GCLC* and *GS* were upregulated, driving de novo *GSH* synthesis to counter the initial oxidative burst. Under severe drought (SD), sustained *GS* expression was complemented by the induction of *PepN*, a peptidase involved in *GSH* salvage, ensuring pool sustainability [47]. This gene−level tuning was mirrored in metabolite dynamics, with precursor accumulation (L−γ−glutamylcysteine) matching biosynthetic demand. Most importantly, this pathway’s activity is linked to a signaling hub: *CIPK6*, another dark−orange module hub gene. *CIPKs* are known to integrate calcium signals, which are early stress messengers [48]. We propose a mechanistic model where stress−induced calcium signals, potentially transduced via *CIPK6*, modulate the transcription and/or activity of *GSH* biosynthetic enzymes, thereby fine−tuning the antioxidant response. This connects early signaling to a definitive metabolic defense output.

The phenylpropanoid pathway exemplifies how alfalfa strategically allocates resources based on stress type. At the gene regulatory level, drought induced a sequential program: initial upregulation of *PAL*, *CHS*, and *DFR* promoted flavonoid/anthocyanin biosynthesis for antioxidant protection [49]; as stress intensified, flux was redirected towards lignin synthesis via marked upregulation of the hub genes *COMT* and *CCR1*. This transcriptional shift caused a measurable metabolic rerouting: accumulation of quercetin and cyanidin under MD/SD, followed by increased provision of monolignol precursors. The physiological consequence is a defense strategy that evolves from chemical quenching (antioxidants) to physical fortification (lignified cell walls) [50,51]. In stark contrast, waterlogging did not strongly activate this flavonoid branch but favored terpenoid accumulation and feruloyl−CoA storage. This indicates a fundamentally different adaptive logic: prioritizing broad−spectrum antimicrobial terpenoids and preparing cell wall reinforcements over producing light−absorbing flavonoids, which may be less beneficial under canopy shade and hypoxia [52].

Phytohormone signaling is pivotal for growth−defense trade−offs. Our data uncover a contrasting hormonal manipulation strategy. Under drought, the significant upregulation of *CKX* (key regulatory gene) directly catalyzes zeatin degradation [53]. This deliberate reduction in active cytokinin levels is a clear metabolic sacrifice of growth−promoting signals, likely to divert resources to defense programs [54]. The concurrent accumulation of O−acetylserine, a cysteine precursor, suggests a coordinated metabolic coupling linking cytokinin catabolism to the bolstering of sulfur−containing antioxidants like GSH [55]. Under waterlogging, however, *CKX* was not induced. Instead, the regulatory strategy shifted to the upregulation of *UGT* genes, which likely glycosylate zeatin to inactive or stored forms. This represents a homeostatic strategy, temporarily inactivating growth signals without irreversible degradation, allowing for quicker recovery post−stress.

**Figure 7:**
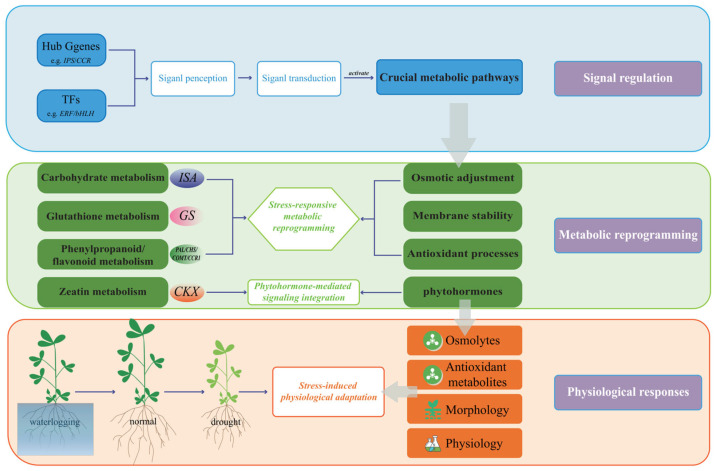
Model diagram illustrating the physiological and molecular mechanisms of alfalfa in response to water stress.

Synthesizing these insights, we propose a refined, mechanistic model for alfalfa’s adaptation to water stress (Figure 7). The model delineates a cause−and−effect cascade:

1. Perception and Signaling (Upstream): Stress−specific signals (e.g., osmotic pressure, hypoxia) are perceived, triggering calcium fluxes and MAPK cascades. Transcription factors (e.g., *ERFs*, *NACs*) and signaling hubs like *CIPK6* are activated.
2. Transcriptional and Metabolic Reprogramming (Intermediate): These regulators drive the targeted expression of key functional genes (*ISA*, *P5CS2*, *GS*, *COMT*, *CCR1*, *CKX*). Their action directly orchestrates metabolic pathway activity, leading to the specific accumulation or depletion of effector metabolites (osmolytes, GSH, flavonoids, lignin precursors, zeatin).
3. Physiological Execution (Downstream): The metabolic changes directly enable physiological outcomes: osmotic adjustment, enhanced ROS scavenging, cell wall lignification, and growth modulation.
4. Morphological Adaptation (Whole−Plant): The integrated physiological state manifests as distinct morphological phenotypes: root architecture remodeling under drought versus root damage and restrained shoot growth under waterlogging.

This model moves beyond correlation to propose testable mechanistic pathways, highlighting specific genes as causal agents (*ISA*, *CIPK6*, *COMT*) in driving the observed multi−omic and phenotypic changes.

This study advances the field by moving from a phenomenological to a mechanistic understanding of alfalfa’s water stress response. Unlike prior omics studies in alfalfa that primarily listed DEGs and DAMs [56,57], our work employs WGCNA to pinpoint functional gene modules and hub genes with high connectivity, proposing their specific roles in causal networks. The innovative integration of physiological traits as “phenotypic anchors” for co−expression network construction directly links molecular modules to tangible plant performance metrics (e.g., the dark−orange module with antioxidant activity).

Furthermore, we provide a comparative mechanistic dissection of drought versus waterlogging responses, revealing not just different gene lists but divergent regulatory logics (e.g., proactive resource reallocation under drought vs. defensive standby under waterlogging). The elucidation of hormonal strategy switching (degradation vs. glycosylation) offers a fresh perspective on how plants fine-tune growth−defense balances under different stresses.

Future work should prioritize functional validation of the proposed hub genes (e.g., *CIPK6*, *COMT*) via genetic approaches and explore the spatial−temporal dynamics of these pathways. Nonetheless, the integrated model presented here provides a robust, mechanistic framework that significantly deepens our understanding of alfalfa adaptation and informs future breeding strategies for climate resilience.

## 4. Materials and Methods

### 4.1. Plant Materials and Experimental Design

In this study, alfalfa cultivar ‘Tamu 1’, bred by Tarim University, was used. Superior individual plants were selected at the early bud stage and propagated vegetatively via stem cuttings to ensure genetic uniformity. During the cutting propagation period, environmental conditions were maintained at a temperature of 25 °C, light intensity of 400 μmol·m^−2^s^−1^, photoperiod of 16 h light/8 h dark, and relative humidity of 75–85%. Plants were grown in plastic pots with an upper diameter of 14 cm, lower diameter of 11 cm, and height of 12 cm. Each pot contained approximately 1.17 kg of a mixed substrate comprising 75% soil and 25% nutrient−rich soil, with a maximum field water capacity (FWC) of approximately 30.13%. When more than 80% of the plants reached a height of 20 cm, a water control experiment was initiated with five treatments: waterlogging (FD, 100 ± 5% FWC), control (CK, 80 ± 5% FWC), light drought (LD, 60 ± 5% FWC), moderate drought (MD, 40 ± 5% FWC), and severe drought (SD, 20 ± 5% FWC). Each treatment included three biological replicates. Soil moisture content was adjusted on days 3, 6, 9, 12, and 15 using the gravimetric method to maintain the target water levels. Plant height was recorded daily between 18:00 and 19:00 h. This fixed late−afternoon sampling time was selected to eliminate diurnal variations in cell turgor and plant expansion driven by transpiration and water uptake, ensuring consistent, comparable, and reproducible phenotypic data across all treatments. The experiment was terminated on day 16 when plants under severe drought began to show wilting symptoms. Leaf samples from all biological replicates of each treatment were immediately wrapped in aluminum foil, frozen in liquid nitrogen, and stored at −80 °C for subsequent metabolomic and transcriptomic analyses.

### 4.2. Measurement of Growth, Physiological, and Biochemical Traits

To assess plant growth and physiological responses, several precise measurements and analyses were performed. Plant height was determined by measuring the straight−line distance from the base of the stem to the apex using a ruler [58], ensuring accuracy in growth tracking. For leaf area analysis, the third fully developed functional leaf from the top was selected and measured using a Yaxin−1241 leaf area meter (Yaxin Instruments, Beijing, China) [59], which provided consistent data on leaf development. Chlorophyll fluorescence parameters were assessed using a PAM−2500 fluorometer (Heinz Walz, Effeltrich, Germany). The third to fourth fully expanded and functional leaves were subjected to a 30 min period of dark adaptation, after which the minimum fluorescence (Fo) and maximum fluorescence (Fm) were recorded. The maximum quantum efficiency of PSII photochemistry was calculated as $\mathrm{Fv}/\mathrm{Fm}=\frac{\left(\mathrm{Fm}-\mathrm{Fo}\right)}{\mathrm{Fm}}$ [60]. While exposed to actinic light, the maximum fluorescence in the light−adapted state (Fm′) was recorded, and the device automatically determined photochemical quenching (qP) and non−photochemical quenching (qN) [27]. Key enzyme activities and stress−related metabolite levels were evaluated using the following techniques: superoxide dismutase (SOD) activity was assessed using the nitroblue tetrazolium (NBT) photoreduction method, peroxidase (POD) activity was measured using the guaiacol method, and catalase (CAT) activity was determined using ultraviolet absorption. Malondialdehyde (MDA) levels were quantified using the thiobarbituric acid (TBA) colorimetric method, and hydrogen peroxide (H_2_O_2_) levels were measured using a commercial assay kit (BC3595, Solarbio, Beijing, China) [61]. Each treatment included three random biological replicates, and all measurements were performed according to this experimental framework. Data are presented as mean ± standard deviation (SD) and were analyzed using SPSS version 27.0. Data analysis was performed using SPSS version 27.0. A one−way ANOVA was utilized, followed by multiple comparisons using Fisher’s Least Significant Difference (LSD) test. Statistical significance was set at *p* < 0.05.

### 4.3. Metabolomics and Statistical Analysis

A sample of alfalfa leaf weighing approximately 50 mg was measured, and 1000 µL of a solution composed of methanol, acetonitrile, and water in a 1:2:1 volume ratio was added. The mixture was vortexed for 30 s, after which a steel bead was introduced. The mixture was then ground at a frequency of 45 Hz for 10 min and subsequently ultrasonicated in an ice−water bath for another 10 min. The sample was stored at −20 °C for an hour before centrifugation at 4 °C at 12,000 rpm for 15 min. An aliquot of 300 µL of the supernatant was filtered through a 0.22 µm organic membrane and collected in a 2 mL injection vial. A 10 µL portion of each sample was combined to create a quality control (QC) sample for instrumental analysis. The analysis utilized a UPLC−ESI−MS/MS system [42], and a Waters HSS−T3 column was used in the process. The mobile phase was composed of pure water with 0.1% formic acid and 5 mM ammonium acetate (Solvent A), along with acetonitrile containing 0.1% formic acid (Solvent B). The samples were analyzed using a gradient elution program that began with 98% A and 2% B for 1.5 min. This was followed by a linear gradient shift to 50% A and 50% B over 5 min, followed by another linear gradient to 2% A and 98% B over 9 min, and held for 1 min. Finally, the conditions were reverted to 98% A and 2% B for 1 min and maintained for 3 min. The column oven was maintained at 50 °C, with a flow rate of 0.35 mL/min and an injection volume of 4 µL. The effluent from the column was alternately linked to an ESI−triple quadrupole linear ion trap (QTRAP) mass spectrometer (MS) for detection. Following data preprocessing and quality evaluation, differential metabolites were identified for further analysis based on the criteria of having a variable importance in projection (VIP) score greater than 1 and a *p*−value less than 0.05.

### 4.4. Transcriptomics and Statistical Analysis

Sequencing was performed using the Illumina NovaSeq 6000 system. The reference genome index was constructed using HISAT2 (v2.0.4) [62], and gene expression levels were adjusted using FPKM [63]. DESeq2 (v1.30.1) was used to perform differential expression analysis between the two groups, identifying differentially expressed genes (DEGs) using the criteria of |log_2_FoldChange| ≥ 1 and FDR < 0.05 [64]. The statistical enrichment of DEGs in KEGG pathways was conducted using clusterProfiler (v4.4.4) along with the KOBAS database. A *p*−value threshold of less than 0.05 was used to determine which KEGG pathways were significantly enriched [65]. Bioinformatics analyses were conducted using the BMKCloud platform. Gene modules that exhibited co−expression were deduced using the R package weighted gene co−expression network analysis (WGCNA) from genes identified in all comparisons with FPKM values exceeding 1. The construction of the network relied on the Topological Overlap Measure (TOM). The soft threshold power (β) was set to 6, the minimum module size was 30 genes, and the module merging cut−off height was 0.25. Eleven phenotypic, physiological, and biochemical traits were chosen as target characteristics to examine the relationships between module eigengenes and these target traits. The co−expression network was visualized using Cytoscape v3.10.3 [47]. The hub genes in the network were identified based on their degree of centrality.

### 4.5. Quantitative Real−Time PCR (qRT-PCR) Validation

RNA was isolated from alfalfa leaf tissue using the TRUEscript RT MasterMix Kit designed for real−time PCR, provided by DF Biotech in Chengdu, China. The purity, concentration, and integrity of the RNA were evaluated using a ScanDrop 100 ultra−micro spectrophotometer (Analytik Jena AG, Jena, Germany). First−strand cDNA was generated from total RNA using the TransScript First−Strand cDNA Synthesis Kit (Aidlab Biotech, Beijing, China). Quantitative real−time PCR (qRT−PCR) was performed using SYBR Green QPCR Mix (DF Biotech, Chengdu, China) on a Bio−Rad CFX Connect Real−Time PCR System (Bio-Rad Laboratories, Hercules, CA, USA). To confirm the RNA−Seq findings, primers for the six candidate genes were designed using Beacon Designer (PREMIER Biosoft, Palo Alto, CA, USA) [66]. Relative gene expression levels were calculated using the 2^−ΔΔCt^ method. The 18S rRNA gene was selected as the stable housekeeping reference gene for expression normalization in qRT−PCR analysis. Primer sequences are listed in Supplementary Table S1.

### 4.6. Statistical Analysis

All experiments were performed in at least three biological replicates. The BMKCloud bioinformatics platform (www.biocloud.net, Origin v2022, and GraphPad Prism 10.6.1 were used to analyze the experimental data. Statistical analysis was conducted using one−way analysis of variance (ANOVA), followed by Fisher’s LSD post hoc test for multiple comparisons. Student’s *t*−test was used to assess significance, with significance levels indicated as * *p* < 0.05, ** *p* < 0.01, and *** *p* < 0.001, and **** *p* < 0.0001.

## 5. Conclusions

This study investigated the phenotypic, physiological, and molecular mechanisms of *Medicago sativa* ‘Tamu 1’ in response to water stress, distinguishing the effects of drought and waterlogging on growth, photosynthesis, and redox balance. Starch and sucrose metabolism, glutathione metabolism, phenylpropanoid/flavonoid biosynthesis, and zeatin biosynthesis were identified as the core stress response pathways. Gene expression showed both conserved and stress−specific patterns: *ISA* and other genes were co−upregulated under both stresses, whereas flavonoid biosynthesis, lignin modification, and cytokinin metabolism exhibited stress−specific regulation. Twenty−eight candidate genes for water stress tolerance were identified, and six key genes were validated by qRT−PCR, laying a foundation for dissecting the adaptation of alfalfa to water stress. Further research is required to characterize their functions and regulatory networks. This study revealed the modular regulatory networks of alfalfa under water stress, enriched the theoretical basis of abiotic stress responses in legumes, and provided valuable gene resources for the molecular breeding of stress−tolerant alfalfa.

## Data Availability

The original contributions presented in this study are included in the article/Supplementary Material. Further inquiries can be directed to the corresponding authors.

## Supplementary Materials

The following supporting information can be downloaded at: https://www.mdpi.com/article/10.3390/plants15101531/s1, Supplementary Figure S1. Metabolomic profiling of alfalfa leaves under water stress. (a) PCA Scores Plot of Metabolites. (b) Heatmap of differentially accumulated metabolites. Supplementary Figure S2. Z-score heatmap of the top 30 differentially accumulated metabolites (DAMs). Supplementary Figure S3. Transcriptomic profiling of alfalfa leaves under water stress. (a) PCA Scores Plot of Transcriptome. (b) Inter-sample correlation heatmap across different water stress treatments. (c) Heatmap of differential differentially expressed genes. Supplementary Figure S4. Weighted gene co-expression network analysis (WGCNA) soft-thresholding power determination for alfalfa leaf transcriptomes under water stress. (a) Heatmap depicting module–sample trait relationships. Each row and column represents a module and a sample trait, respectively. Supplementary Table S1. Statistics of Sequencing Data. Supplementary Table S2. Transcription factor family distribution in key WGCNA modules associated with different water stress treatments in alfalfa.Supplementary Table S3. List of metabolites exhibiting significant changes (relative content) in key metabolic pathways under water stress. Supplementary Table S4. Relative content of proline under water stress. Supplementary Table S5. List of genes exhibiting significant changes (FPKM) in key metabolic pathways under water stress.
