# Supplementary material for: Integrated Transcriptomic and Metabolomic Analyses Reveal Adaptive Mechanisms of Medicago sativa Under Water Stress
Source: Plants (Basel). 2026 May 16;15(10):1531. doi: 10.3390/plants15101531 (PMC13211047; doi:10.3390/plants15101531)

Supplementary Figure S2. Z-score heatmap of the top 30 differentially accumulated metabolites (DAMs).

(a)

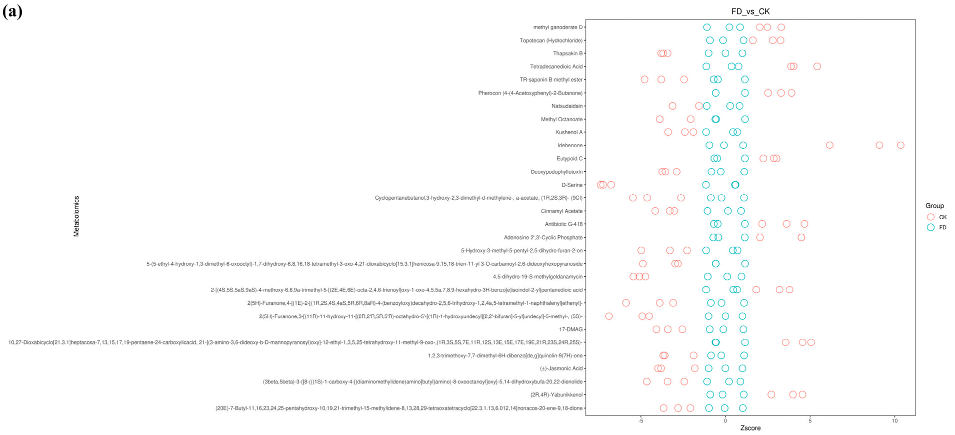

(b)

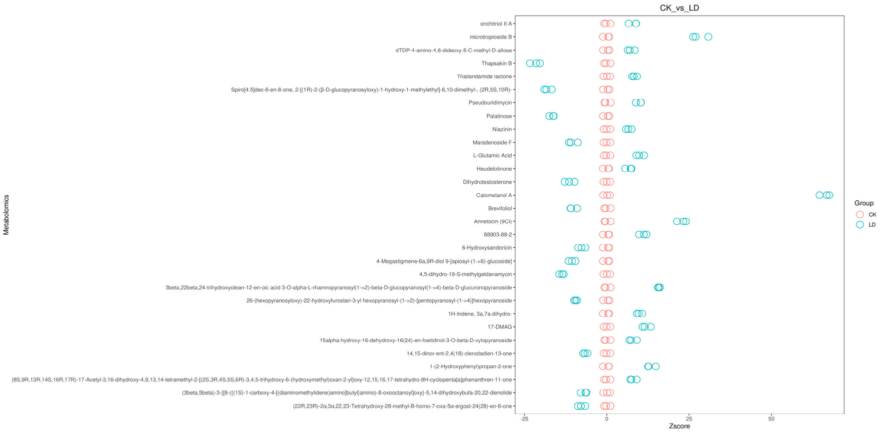

(c)

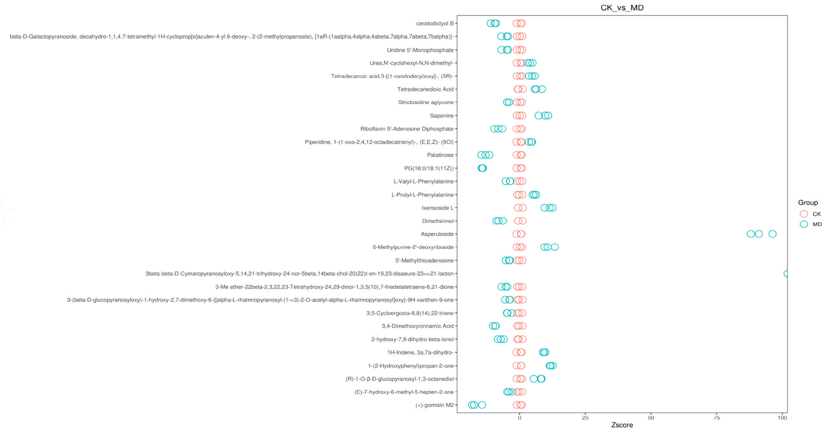

(d)

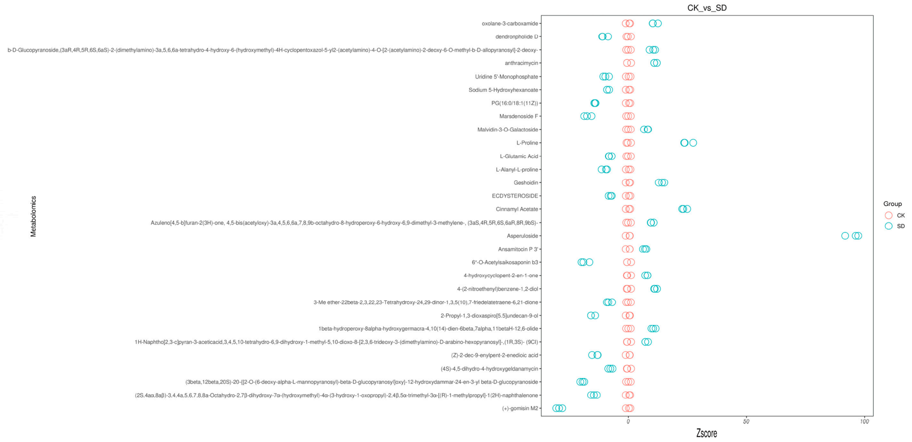

Supplement: Supplementary file 1 [file plants-15-01531-s001.zip › Supplementary Figure S2.pdf]
