# Supplementary material for: Integrated Transcriptomic and Metabolomic Analyses Reveal Adaptive Mechanisms of Medicago sativa Under Water Stress
Source: Plants (Basel). 2026 May 16;15(10):1531. doi: 10.3390/plants15101531 (PMC13211047; doi:10.3390/plants15101531)

Supplementary Figure S3. Transcriptomic profiling of alfalfa leaves under water stress. (a) PCA Scores Plot of Transcriptome. (b) Inter-sample correlation heatmap across different water stress treatments. (c) Heatmap of differential differentially expressed genes.

(a)

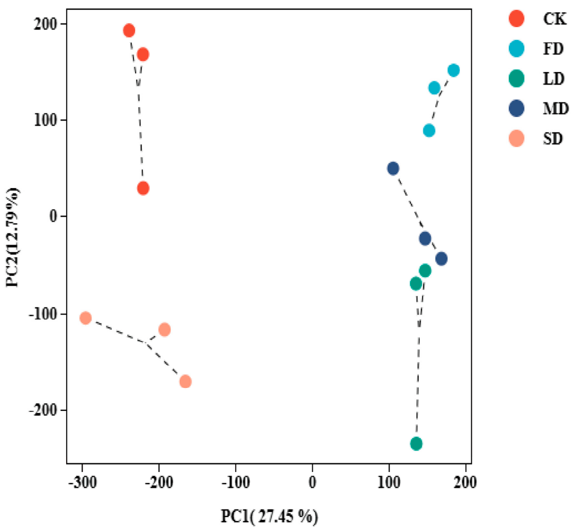

(b)

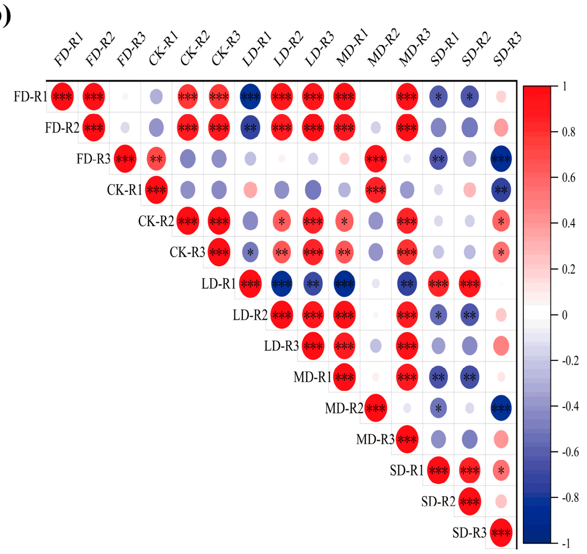

(c)

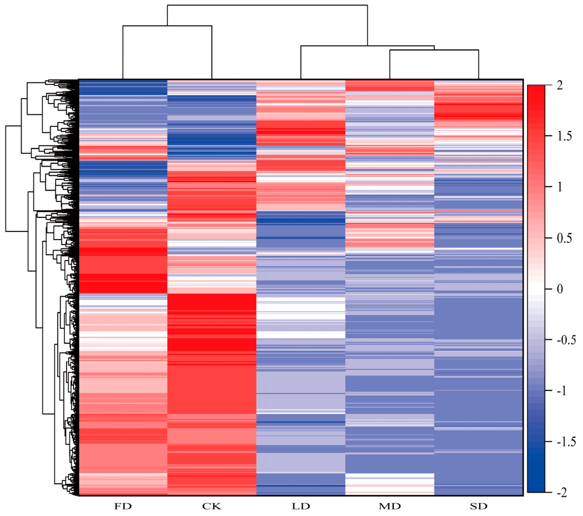

Supplement: Supplementary file 1 [file plants-15-01531-s001.zip › Supplementary Figure S3.pdf]
