# Supplementary material for: Integrated Transcriptomic and Metabolomic Analyses Reveal Adaptive Mechanisms of Medicago sativa Under Water Stress
Source: Plants (Basel). 2026 May 16;15(10):1531. doi: 10.3390/plants15101531 (PMC13211047; doi:10.3390/plants15101531)

Supplementary Figure S4. Weighted gene co-expression network analysis (WGCNA) soft-thresholding power determination for alfalfa leaf transcriptomes under water stress. (a) Heatmap depicting module - sample trait relationships. Each row and column represents a module and a sample trait, respectively.

(a)

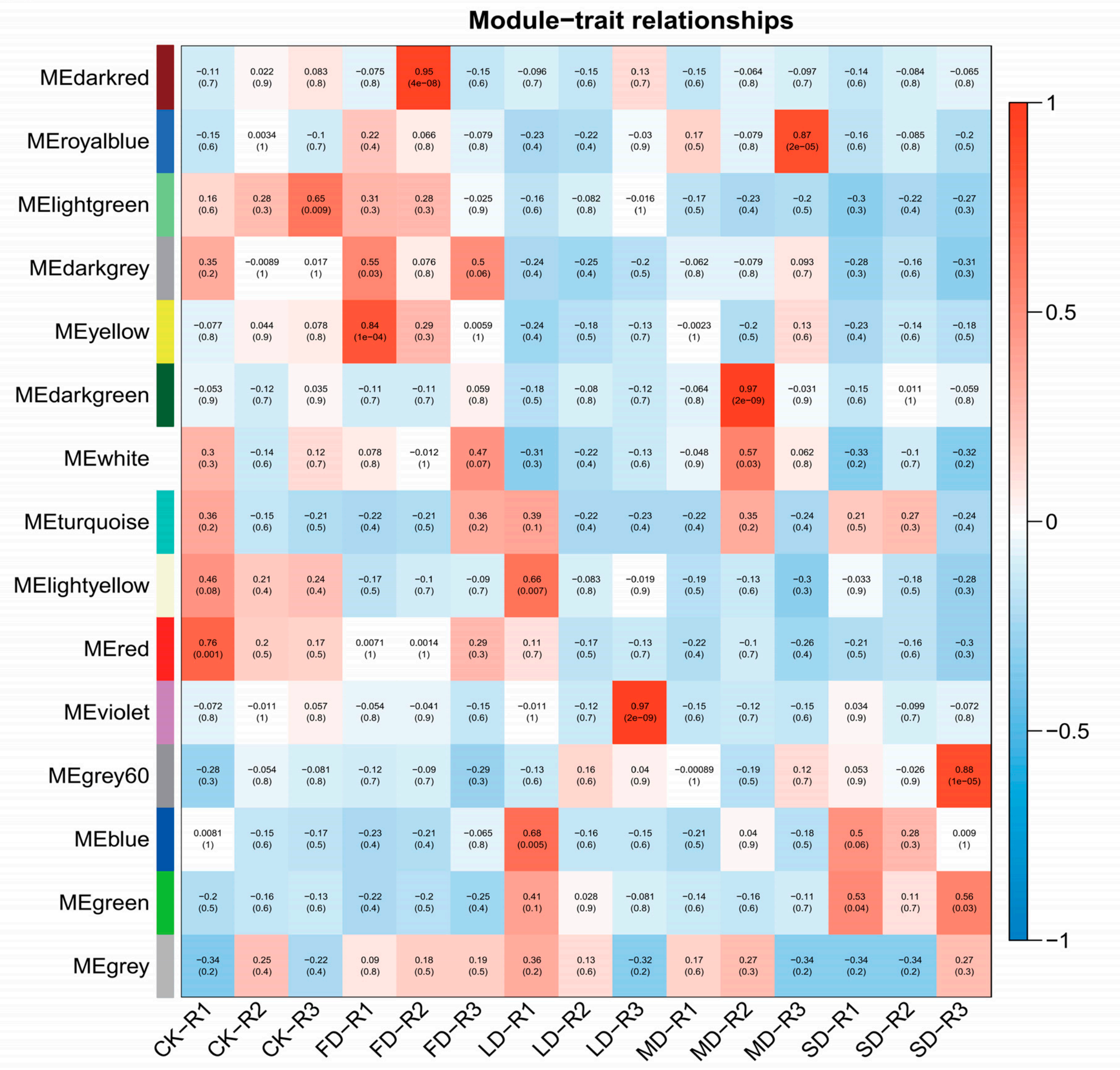

Supplement: Supplementary file 1 [file plants-15-01531-s001.zip › Supplementary Figure S4.pdf]
