# Supplementary material for: Integrated Transcriptomic and Metabolomic Analyses Reveal Adaptive Mechanisms of Medicago sativa Under Water Stress
Source: Plants (Basel). 2026 May 16;15(10):1531. doi: 10.3390/plants15101531 (PMC13211047; doi:10.3390/plants15101531)
Supplement: Supplementary file 1 [file plants-15-01531-s001.zip › Supplementary Table S1.pdf]

Supplementary Table S1. Statistics of Sequencing Data

SampleID	ReadSum	BaseSum	GC(%)	N(%)	Q20(%)	Q30(%)
CK-R1	2.25E+07	6.66E+09	41.99	0.01	97.55	93.13
CK-R2	2.39E+07	7.11E+09	41.86	0.01	97.67	93.64
CK-R3	2.28E+07	6.76E+09	41.85	0.01	97.49	93.15
FD-R1	2.47E+07	7.36E+09	41.94	0.01	97.7	93.61
FD-R2	2.32E+07	6.88E+09	42.03	0.01	97.54	93.13
FD-R3	2.13E+07	6.30E+09	42.31	0.01	97.51	93.11
LD-R1	2.50E+07	7.46E+09	41.74	0.01	97.66	93.51
LD-R2	2.24E+07	6.66E+09	41.97	0.01	97.59	93.27
LD-R3	2.25E+07	6.67E+09	41.97	0.01	97.53	93.2
MD-R1	2.40E+07	7.12E+09	42.13	0.01	97.61	93.3
MD-R2	2.17E+07	6.41E+09	42.48	0.01	97.52	93.05
MD-R3	2.41E+07	7.15E+09	42.19	0.01	97.69	93.68
SD-R1	2.25E+07	6.69E+09	41.96	0.01	97.6	93.42
SD-R2	2.45E+07	7.28E+09	41.72	0.01	97.6	93.39
SD-R3	2.64E+07	7.88E+09	41.98	0.01	97.74	93.77
